# (-)-Epigallocatechin gallate attenuates NADPH-d/nNOS expression in motor neurons of rats following peripheral nerve injury

**DOI:** 10.1186/1471-2202-12-52

**Published:** 2011-06-01

**Authors:** I-Hua Wei, Hui-Chin Tu, Chih-Chia Huang, Mang-Hung Tsai, Chi-Yu Tseng, Jeng-Yung Shieh

**Affiliations:** 1 Department of Anatomy and Cell Biology, College of Medicine, China Medical University, Taichung, Taiwan; 2 Department of Psychiatry, China Medical University and Hospital, Taichung, Taiwan; 3 Department of Neurology, Taichung Tzu Chi General Hospital, Taichung, Taiwan; 4 Department of Anatomy and Cell Biology, College of Medicine, National Taiwan University, Taipei, Taiwan

## Abstract

**Background:**

Oxidative stress and large amounts of nitric oxide (NO) have been implicated in the pathophysiology of neuronal injury and neurodegenerative disease. Recent studies have shown that (-)-epigallocatechin gallate (EGCG), one of the green tea polyphenols, has potent antioxidant effects against free radical-mediated lipid peroxidation in ischemia-induced neuronal damage. The purpose of this study was to examine whether EGCG would attenuate neuronal expression of NADPH-d/nNOS in the motor neurons of the lower brainstem following peripheral nerve crush. Thus, young adult rats were treated with EGCG (10, 25, or 50 mg/kg, i.p.) 30 min prior to crushing their hypoglossal and vagus nerves for 30 seconds (left side, at the cervical level). The treatment (pre-crush doses of EGCG) was continued from day 1 to day 6, and the animals were sacrificed on days 3, 7, 14 and 28. Nicotinamide adenine dinucleotide phosphate-diaphorase (NADPH-d) histochemistry and neuronal nitric oxide synthase (nNOS) immunohistochemistry were used to assess neuronal NADPH-d/nNOS expression in the hypoglossal nucleus and dorsal motor nucleus of the vagus.

**Results:**

In rats treated with high dosages of EGCG (25 or 50 mg/kg), NADPH-d/nNOS reactivity and cell death of the motor neurons were significantly decreased.

**Conclusions:**

The present evidence indicated that EGCG can reduce NADPH-d/nNOS reactivity and thus may enhance motor neuron survival time following peripheral nerve injury.

## Background

Peripheral nerve injury (PNI) produces numerous morphological, physiological, and biosynthetic changes in the damaged neurons [1-3]. Damage caused by PNI is associated with a variety of mechanisms, especially oxidative stress and subsequent massive production of oxygen-derived free radicals, which are thought to play a major role in neurological disorders [4]. First identified as the endothelium-derived relaxing factor [5,6], nitric oxide is a short-lived free radical and gaseous biological messenger produced by nitric oxide synthase (NOS). This enzyme catalyzes the oxidation of L-arginine and nicotinamide adenine dinucleotide phosphate (NADPH) by O_2 _to yield L-citrulline and NO [7]. Three distinct NOS isoforms have been described: inducible (iNOS), endothelial (eNOS), and neuronal (nNOS) [8]. In the nervous system, free radical gas plays an important role in the regulation of physiological activities ranging from synaptic plasticity to neuroendocrine functions [9]. NO combines rapidly with superoxide to form peroxynitrite, a potent oxidizing agent with cytotoxic actions [10-12]. Previous studies have indicated that PNI in adult animals results in dramatic up-regulation of nitric oxide synthase in certain types of central and peripheral neurons that normally lack or hardly ever express the enzyme [13-18]. The injury-induced expression of NOS seems to signal the impending death of lesioned cells, as the enzyme acts as a killer protein by producing neurotoxic levels of NO [19-28]. Accordingly, numerous studies have examined, in various models of injury, the possibility of oxygen free radical scavengers as therapeutic agents for oxidative neuronal damage [22,26,29].

Green tea (*Camellia sinensis*) is one of the most popular beverages in the world because of its attractive flavor and aroma. It contains an abundance of so-called 'green tea polyphenols (GTPs), typically flavonoids or catechins, which are polyphenolic complexes with potential free radical scavenger activity. Many studies have shown that GTP can scavenge reactive oxygen species (ROS) such as hydroxyl radical (·OH), hydrogen peroxide (H_2_O_2_), and superoxide anion (O_2_^-^) as well as reactive nitrogen species (RNS) such as nitric oxide (·NO) and peroxynitrite (ONOO^-^) in many systems. GTP also acts to protect cells from damage induced by the free radicals in the gaseous phase [30-35]. Green tea contains many polyphenolic compounds, including (-)-epigallocatechin gallate (EGCG), (-)-epigallocatechin (EGC), (-)-epicatechin gallate (ECG), and (-)-epicatechin (EC). Of these, EGCG is the most abundant and with two triphenolic groups, it is the most effective antioxidant [36,37]. Although the mechanisms of EGCG's antioxidant activity remain unclear, pharmacological studies have identified several antioxidant properties such as: (1) blockade of nNOS and iNOS induction [34,38,39] and (2) scavenging of free radicals or attenuation of lipid peroxidation [36,37,40]. Thus, EGCG is expected to be neuroprotective.

To our knowledge, the potential effects of EGCG on NOS activity and neuronal damage following nerve injury *in vivo *remain to be explored. Notably, most previous studies concerning the effects of EGCG on NOS activity are biochemical rather than morphological. Thus, we carried out a morphological study using nicotinamine adenine dinucleotide phosphate-diaphorase (NADPH-d) histochemistry along with nNOS immunohistochemistry to explore the time-course of nNOS expression in the hypoglossal nucleus (HN) and dorsal motor nucleus (DMN) of the vagus in rats subjected to peripheral nerve crush injury (PNCI) and to elucidate the effect of post-PNCI EGCG treatment on motor neurons in the lower brainstem.

## Methods

### Treatment of experimental animals

Adult male Wistar rats (n = 80) weighing 200 - 250 g were used and obtained from the Laboratory Animal Center of the National Taiwan University. We followed the Guide for the Care and Use of Laboratory Animals (1985) as stated in the United States NIH guidelines (NIH publication no.86-23) to treat animals for this study including the care, housing, handling and experimental procedures. All the experiments were approved by our Laboratory Animal Center, China Medical University, Taiwan. All efforts were made to minimize both the animal suffering and the number of animals used for this investigation.

The experimental animals were anesthetized with an intramuscular (i.m.) injection of mixtures of zoletil (30 mg/kg) and xylazine (10 mg/kg). After anesthesia, the left vagus and the hypoglossal nerves were subjected to crush injury by clamping with a small hemostatic forceps for 30 s; for the former, the level of clamping was mid-cervical, whereas for the latter, right below the tendon of the digastric muscle. In sham-operated animals (controls), similar procedures were carried out except both of the left side nerves remained intact. The experimental animals were divided into four groups: I, II, III, and IV (10, 25, 50 mg/kg EGCG-pretreatment + PNCI and PNCI only, respectively). Previous *in vivo *studies have demonstrated that EGCG can pass the blood-brain barrier and reach the brain parenchyma [34,38,41,42]. Animals in each group were therefore received daily intraperitoneal injections of EGCG or normal saline for successive six days with the last injection at 30 min before PNCI. Different doses (10, 25, or 50 mg/kg body weight dissolved in normal saline) of EGCG (cat No.E4143, Sigma-Aldrich, St Louis, MO, USA) were applied in the present study. Each experimental group was subdivided into four groups (n = 5) and sacrificed at 3, 7, 14, and 28 days. All the experimental animals were housed under the same conditions (controlled temperature [22°C] and 12-h light/dark cycle) and given free access to food and water *ad libitum*.

### Perfusion and tissue preparation

At each time point, rats were deeply anesthetized with mixtures of zoletil (30 mg/kg) and xylazine (10 mg/kg) and perfused transcardially with 100 ml of Ringer's solution followed by 300 ml of 4% paraformaldehyde in 0.1 M phosphate buffer (PB), pH 7.4. After perfusion, the brainstem and its nuclei were quickly removed, rinsed in 0.1 M PB, and transferred through a series of increasing concentrations of sucrose buffer (10 - 30%) for cryoprotection at 4°C overnight. Serial 30-μm thick sections of the brainstem were cut transversely with a cryostat on the following day and were alternately placed at a distance of 120 μm apart into four-well dishes. Sections collected in the first and second wells were processed for NADPH-d histochemistry and counterstained with neutral red; sections in the third well were processed for nNOS immunohistochemistry and those in the fourth well were processed for nNOS immunofluorescence labeling combined with NADPH-d histochemistry.

### NADPH-d histochemistry

NADPH-d used as a selective marker for nNOS as described previously [21,27,43,44]. Briefly, sections in the first two wells were incubated with NADPH-d medium (0.1 mg/ml nitroblue tetrazolium, 1 mg/ml β-NADPH, and 0.3% Triton X-100 in 0.1 M PB [pH 7.4]) for 1 h at 37°C, washed several times in 0.1 M PB to terminate the reaction. For counting neuronal numbers, sections from the second well were further counterstained with neutral red, dehydrated through a graded series of alcohols, cleared with xylene, and coverslipped with Permount.

### nNOS immunohistochemistry

Sections in the third well were rinsed in 0.01 M phosphate buffered saline (PBS), pH 7.4, treated with 0.01 M PBS containing 10% methanol and 3% hydrogen peroxide for 1 h to abolish the endogenous peroxidase activity, rinsed 3 times with PBS, and incubated with medium containing 3% normal horse serum, 2% bovine serum albumin, and 0.1% Triton X-100 for 1 h. The reacted sections were then washed several times with PBS, incubated with antibody to nNOS (1:100; Santa Cruz Biotechnology, Burlingame, CA, USA) for 24 h at 4°C, treated with biotinylated secondary antibody (1:200; Vector Laboratories, Burlingame, CA, USA) for 1 h at room temperature, and incubated with Streptavidin/HRP (DAKO A/S, Glostrup, Denmark). The signal was developed with diaminobenzidine (a peroxidase substrate).

### Colocalization of nNOS and NADPH-d reactivity

Colocalization of NADPH-d and nNOS was detected by immunofluorescence labeling of nNOS followed by histochemical staining for NADPH-d [21,27,43,44]. Sections in 4-well dishes were rinsed in 0.01 M PBS, incubated in medium containing 3% normal goat serum, 2% bovine serum albumin, and 0.1% Triton X-100 for 1 h to block nonspecific binding, rinsed with PBS, and incubated with mouse monoclonal antibody against nNOS (1:100; Santa Cruz Biotechnology) for 24 h at 4°C. The reacted sections were then treated with fluorescein isothiocyanate (FITC)-conjugated anti-rabbit secondary antibody (1:200; Vector Laboratories) for 2 h at room temperature, washed several times with PBS, and coverslipped with buffered glycerin to prevent fading. The tissue slides were photographed using a ZEISS fluorescence microscope equipped with an appropriate excitation filter (450-490 nm) for observing FITC-labeled nNOS-immunoreactive neurons. After photographing, the sections were again washed several times again in 0.1 M PB, processed for NADPH-d histochemistry (as described above), rapidly dehydrated through a graded series of alcohols, cleared with xylene, and coverslipped with Permount.

### Quantitative study and image analysis

In each animal, 20-30 sections representing the entire length of the HN and DMN (extending from the obex 1.8 mm caudally and 1 mm rostrally) were collected for NADPH-d histochemistry and neutral red counterstaining and cell counting. To avoid bias for the counts, large neurons (25-50 μm) with clearly outlined nuclei but not small motor neurons were counted due to the latter could not be distinguished from interneurons being 10-18 μm in diameter. Both the NADPH-d positive and negative (neutral red-counterstained) cells in the HN and DMN, either ipsilateral or contralateral to the nerve crush site, were counted and summed to present the total number of cells in 20-30 sections (unit volume, u). The labeling percentage was calculated by dividing the number of NADPH-d(+) neurons on the lesion side by the total number of neurons on the same side. To confirm the coexistence of NADPH-d(+) and nNOS(+) neurons, double-labeled neurons in the HN and DMN of the same sections were counted on the photomicrographs (data not shown). Cells containing nNOS were counted in the sections processed for NADPH-d histochemistry since neuronal NADPH-d is a reliable marker of nNOS [21,43]. Besides, the results of neuronal staining in sections stained for NADPH-d are usually better than nNOS, which in turn facilitates computer-assisted quantitative assessment. Labeling intensity of NADPH-d(+) neurons was quantified using a computer-based image analysis system (MGDS) and Image Pro-Plus software (Media Cybernetics, Silver Spring, MD, USA). Labeled sections were scanned at 100 × magnification in bright field using a digital camera mounted on a ZEISS microscope and the images were displayed on a high-resolution monitor. Up to 100 cells per section were measured along the entire length of the HN and DMN. At 100 × magnification, the cytoplasmic optical density (OD), which was used as an index of labeling intensity, in NADPH-d(+) neurons was measured by tracing the contour of the labeled soma in digitized images. The background OD in each section was measured by averaging five random polygons (area of polygon = 150 μm^2^) within the neuropil of the corresponding HN and DMN regions on the non-lesioned side. The staining intensity in a tissue section reflected the amount of enzyme activity. Thus, all parameters were controlled using methods to ensure consistent gray level adjustment, histogram stretch, and minimal optical density. To avoid introducing bias, two observers blinded to the animal treatment group counted cells in the NADPH-d stained sections and evaluated the data from image analysis of HN and DMN neurons.

### Statistical analysis

All data of this study were expressed as mean ± SEM and statistical significance was determined with a commercially available software package (SPSS version 12; SPSS, Chicago, IL). Between-group differences in the percentage and OD of NADPH-d positive neurons at various time points, with or without EGCG treatment, were evaluated using one-way analysis of variance (ANOVA) followed by LSD post hoc test. The data collected in experimental groups within the same time point (i.e., 10 mg/kg EGCG pretreated versus control non-treated group) were further analyzed using Student's *t *test. Statistical difference was considered significant if *p *< 0.05.

### Control experiments

Some negative controls were included to ensure the validity of the NADPH-d histochemical and nNOS immunohistochemical results. Thus, the medium without β-NADPH was used as the control in NADPH-d staining protocol, whereas media without primary or secondary antibodies were used as controls in nNOS immunochemical procedure.

## Results

### NADPH-d/nNOS colocalization studies

**Figure 1:**
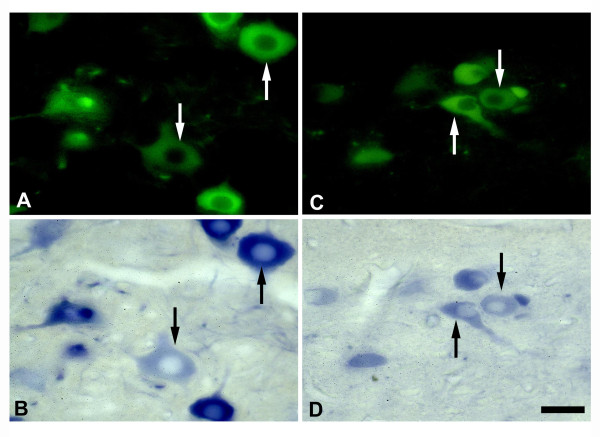
**NADPH-d/nNOS colocalization studies**. Fluorescence (A, C) and light (B, D) photomicrographs showing nNOS(+) (A, C) and NADPH-d(+) (B, D) labeled neurons on the lesioned side of the HN (A, B) and DMN (C, D) of rats following crush injury of left vagus and hypoglossal nerves. Note that a most of nNOS(+) neurons (arrows in A-D) contain NADPH-d in the HN and DMN. Scale bar = 50 μm.

Labeling for both nNOS and NADPH-d showed that the majority of the NADPH-d positive neurons in the HN and DMN were also positive for nNOS (Figure 1). The distribution of nNOS-positive neurons in the brainstem motor nuclei paralleled that of NADPH-d positive neurons. The time course of PNCI-induced nNOS expression in the HN and DMN on the lesion side coincided with that of PNCI-induced NADPH-d expression. Since the NADPH-d histochemical method was sensitive and specific, it was used for both qualitative and quantitative analyses. No NADPH-d and nNOS positive neurons were detected in the HN or the DMN of negative control rats.

### NADPH-d/nNOS expression in the hypoglossal nucleus of EGCG-treated rats following PNCI

**Figure 2:**
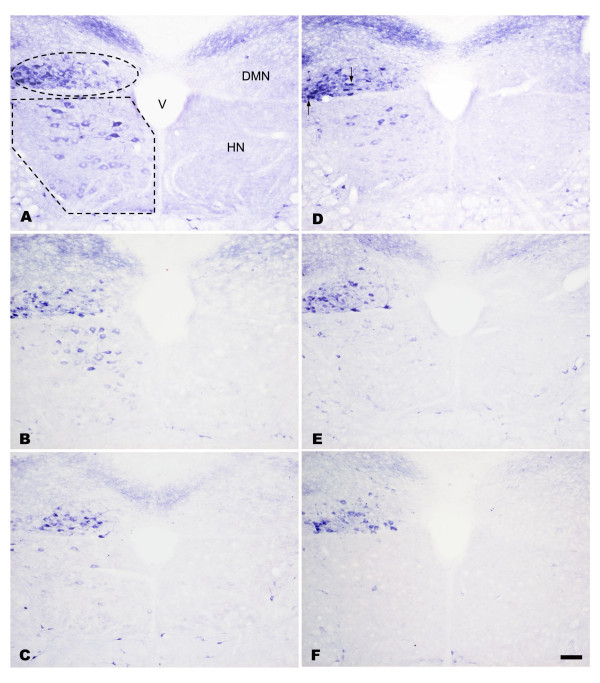
**Histochemistry of NADPH-d in the HN and DMN**. Light photomicrographs showing the injury-induced NADPH-d reactivity in the lesioned side of the HN and DMN at 7 (A, B, C) and 14 (D, E, F) days after PNCI. NADPH-d staining is barely detected in HN and DMN neurons in the contralateral intact side of the nuclei (the right side of each figure). Note the marked increase in NADPH-d reactivity both in terms of staining intensity and number of labeled neurons at 7 days in the HN and DMN after PNCI (A, B, C). Also note that the population of NADPH-d(+) neurons in the HN is reduced at 14 days after PNCI, whereas that in the DMN tends to increase (D, E, F) when compared those at 7 days post PNCI. Some shrunken cells (D, arrows) are found to display intense NADPH-d reactivity. In rats receiving EGCG treatment at high dose (50 mg/kg, C, F), the diaphorase reactivity and the number of positive neurons are noticeably reduced in relation to those of non-treated rats (A, D) or animals treated with lower dose EGCG (10 mg/kg, B, E). The ellipse and polygonal dotted line in A outlines the area of the HN and DMN, respectively. V, fourth ventricle; Scale bar = 100 μm.

**Figure 3:**
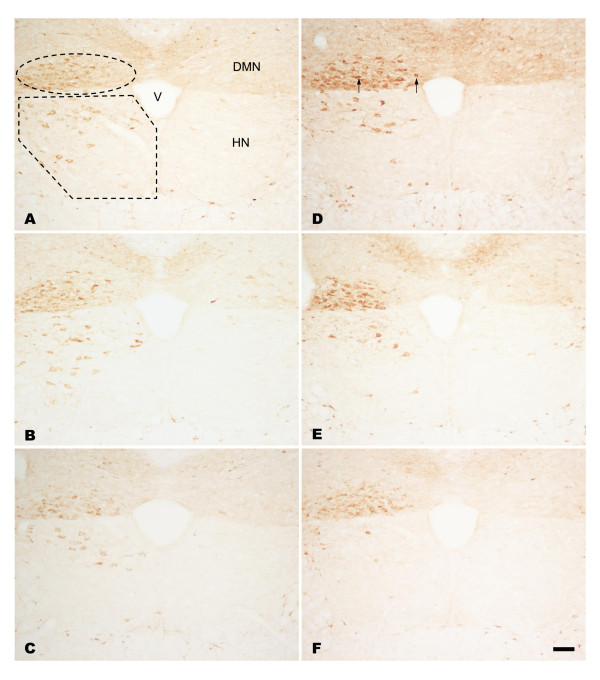
**Immunohistochemistry of nNOS in the HN and DMN**. Light photomicrographs showing the nNOS reactivity in the lesioned side of the HN and DMN at 7 (A, B, C) and 14 (D, E, F) days after PNCI. nNOS staining is barely detected in HN and DMN neurons in the contralateral intact side of the nuclei (the right side of each figure), but significantly increased at 7 days in the affected HN and DMN after PNCI (A, B, C). When compared with those at 7 days post PNCI, nNOS positive neurons in the HN are obviously decreased in number at 14 days following PNCI, whereas those in the DMN are inclined to increase (D, E, F). The immunoreactivities of nNOS are markedly declined in the lesioned HN and DMN of rats treated with high dose EGCG (50 mg/kg, C, F) in relation to those of non-treated rats (A, D) or animals treated with lower dose EGCG (10 mg/kg, B, E). Similarly, there were some nNOS intensely-labeled neurons appear shrunken in contour (D, arrows) following PNCI. V, fourth ventricle; Scale bar = 100 μm.

**Figure 4:**
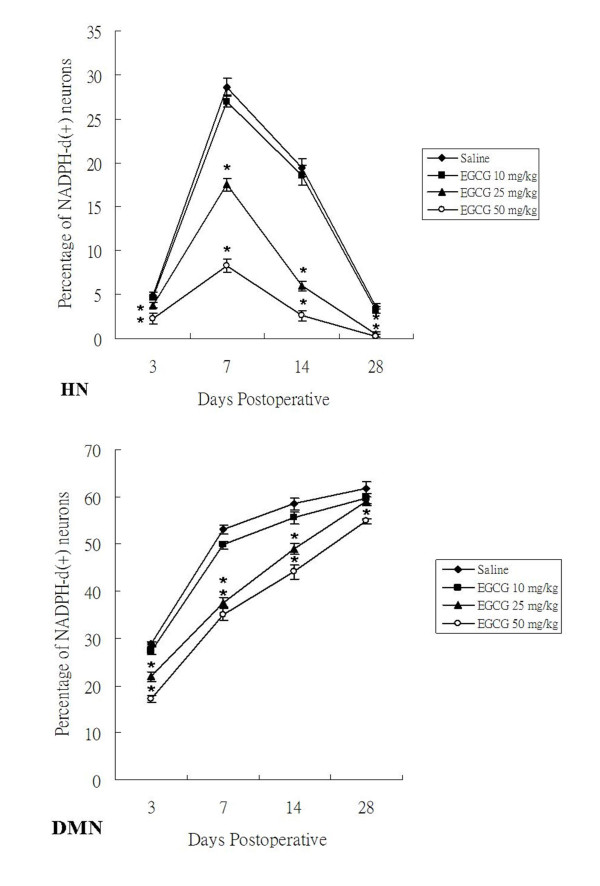
**Alterations of NADPH-d reactivity in the HN and DMN at 3, 7, 14 and 28 days after PNCI and EGCG treatment**. Quantitative analysis shows that PNCI causes a remarkably-increased population of NADPH-d(+) neurons at different time intervals examined. PNCI-induced NADPH-d(+) neurons reach their population peak at 7 days in the HN but continue to increase their population in the DMN until the end of the examination. Note a significant decrease of NADPH-d(+) neurons in the groups receiving EGCG treated animals when compared with those of non-treated (saline-treated) ones at 7 and 14 days in the HN. At the same time intervals in the DMN, the groups receiving high dosages of EGCG (25 and 50 mg/kg) show a marked reduction of NADPH-d(+) neuron population when compared with those receiving lower dose of EGCG (10 mg/kg) or saline. *, *p *< 0.05 for the comparison between EGCG-treated and saline-treated groups at the same survival time point.

**Figure 5:**
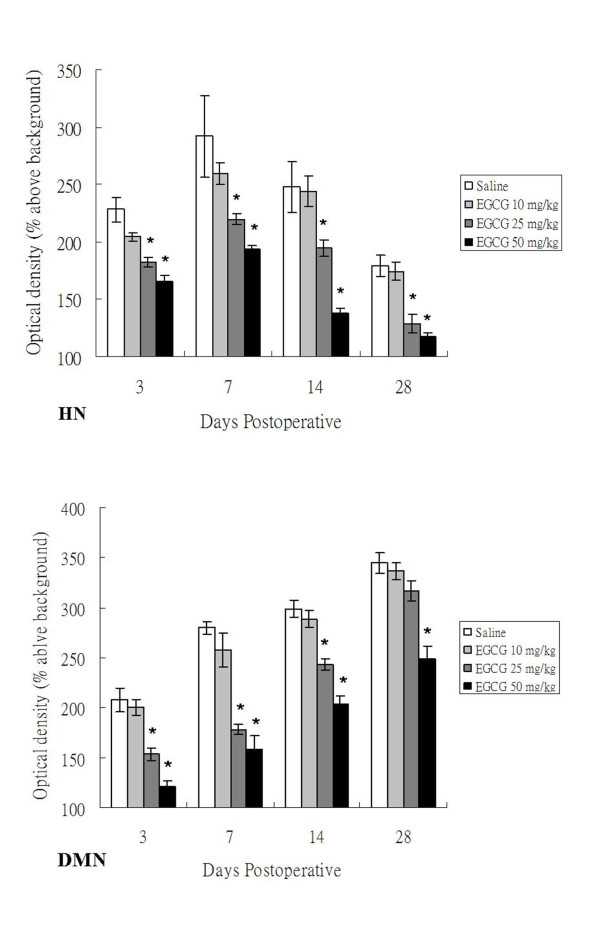
**Mean optical density (OD) of NADPH-d(+) neurons in the HN and DMN of adult rats at 3, 7, 14 and 28 days after PNCI and EGCG treatment**. The changes of mean optical density (determining staining intensity) in NADPH-d(+) neurons of examined nuclei show a similar trend to the population modification of labeled neurons. The OD value in the HN of high dose EGCG-treated (25 and 50 mg/kg) animals is much lower than those of low dose EGCG-treated (10 mg/kg) or non-treated ones at 7, 14 and 28 days after PNCI. The EGCG-mediated noticeable decrease of NADPH-d optical density is also evidenced in the DMN at different time points after PNCI. *, *p *< 0.05 for the comparison between EGCG-treated and saline-treated groups at the same survival time point.

In sham-operated groups of both non-treated and EGCG-treated rats, no NADPH-d/nNOS positive [NADPH-d/nNOS(+)] neurons were found on either side of the hypoglossal nucleus (HN). In non-treated rats subjected to PNCI, NADPH-d/nNOS(+) neurons were first detected in the HN at 3 days, with their number reaching a peak at 7 days (*p *< 0.05 vs. various time points; Figures 2A, 3A and 4; Table 1). The percentage of NADPH-d/nNOS(+) neurons increased steeply from 4.92 ± 0.29% at 3 days to 28.64 ± 0.93% at 7 days after PNCI (Figure 4) but then declined to 19.45 ± 0.95% and 3.69 ± 0.28% at 14 and 28 days, respectively, in rats with longer survival times (Figures 2D, 3D and 4). It is also true for the changes of cell total number that showed an increased population of cells at various time points and reached the climax at 7 days (370 ± 9 cell/u; Table 1) after PNCI. The distribution of NADPH-d/nNOS(+) neurons was random (Figures 2 and 3) and, at 3 days after PNCI, the staining intensity of most labeled neurons was weak, but gradually enhanced to 292 ± 35% above background values at 7 days following PNCI (Figure 5). At the same time, shrunken NADPH-d intensely-labeled neurons occurred (Figures 2 and 3).

**Table 1:** Effect of EGCG treatment on the total number of NADPH-d positive (+) and negative (-) cells in the lesioned side of DMN and HN at 3, 7, 14 and 28 days following PNCI

Days postoperative	Hypoglossal nucleus							
	
	Saline		EGCG (10 mg/kg)		EGCG (25 mg/kg)		EGCG (50 mg/kg)	
	
	+	-	+	-	+	-	+	-
3	69 ± 4	1336 ± 7	65 ± 3	1343 ± 7	53 ± 4	1344 ± 9	31 ± 4*	1368 ± 7*
7	370 ± 9	924 ± 22	352 ± 11	952 ± 11	238 ± 10*	1121 ± 11*	114 ± 10*	1251 ± 16*
14	235 ± 9	963 ± 34	229 ± 11	1009 ± 29	79 ± 8*	1239 ± 10*	35 ± 7*	1296 ± 12*
28	43 ± 3	1122 ± 19	38 ± 4	1137 ± 31	6 ± 4*	1284 ± 6*	4 ± 3*	1297 ± 5*

**Days postoperative **	**Dorsal motor nucleus of vagus nerve**							
	
	**Saline**		**EGCG (10 mg/kg)**		**EGCG (25 mg/kg)**		**EGCG (50 mg/kg)**	
	
	+	-	+	-	+	-	+	-

3	368 ± 9	909 ± 6	351 ± 11	934 ± 8	283 ± 12*	1009 ± 18*	222 ± 9*	1067 ± 10*
7	628 ± 21	554 ± 3	590 ± 14	595 ± 9	462 ± 13*	765 ± 17*	433 ± 18*	802 ± 13*
14	616 ± 12	439 ± 20	599 ± 13	480 ± 17	580 ± 11	603 ± 19*	525 ± 17*	667 ± 18*
28	588 ± 16	363 ± 15	586 ± 11	397 ± 14	603 ± 14	418 ± 12*	608 ± 24	482 ± 17*

*, *p *< 0.05 when compared with the saline-treated group

In rats receiving high dosages of EGCG (25 and 50 mg/kg) treatment then subjected to PNCI, their staining pattern of NADPH-d/nNOS was paralleled to those of non-treated ones, but their total number and percentage of the labeled neurons were comparatively lower than those of non-treated groups (*p *< 0.05, Figures 2, 3 and 4, Table 1). Similarly, EGCG-treated (25 and 50 mg/kg) rats at various time points after PNCI showed a marked increase in the total number of NADPH-d negative cells (*p *< 0.05 vs. non-treated groups; Table 1). The percentage of NADPH-d/nNOS(+) neurons was slightly increased from 3.82 ± 0.27% and 2.26 ± 0.65% at 3 days to 17.52 ± 0.70% and 8.33 ± 0.76% at 7 days respectively in EGCG-treated (25 and 50 mg/kg) rats after PNCI (Figure 4). In EGCG-treated rats killed at longer survival periods after PNCI, the percentage of NADPH-d/nNOS(+) neurons was gradually decreased at 14 days and approached to zero at 28 days (Figure 4). EGCG treatment did not alter NADPH-d/nNOS(+) neuron distribution but statistical evaluation revealed that the staining intensity of NADPH-d/nNOS appeared to be lower in the treated groups than non-treated ones at all time intervals post-injury (*p *< 0.05, Figure 5). Low-dose (10 mg/kg) EGCG treatment had no effect on enzyme amount and expression (Figures 2B, D, 3B, D, 4 and 5; Table 1).

### NADPH-d/nNOS expression in the dorsal motor nucleus of vagus of EGCG treated rats following PNCI

In sham-operated groups of the rats treated with or without EGCG, hardly any detected NADPH-d/nNOS(+) neurons were found either on the left or right side of the DMN. From 3 days after PNCI, NADPH-d/nNOS(+) neurons were detected and randomly distributed throughout the injured DMN (Figures 2 and 3). There were some shrunken cells expressing intense NADPH-d at 14 days following PNCI (Figures 2 and 3). At the same time, treatment with high doses of EGCG (25 and 50 mg/kg) tended to decrease the total number and percentage of NADPH-d/nNOS(+) neurons in the DMN (462 ± 13 cell/u; 37.62 ± 1.09% and 433 ± 18 cell/u; 35.06 ± 1.31%, respectively) when compared with the non-treatment one (628 ± 21 cell/u; 53.08 ± 0.91%; Figure 4 and Table 1). The total number of NADPH-d negative cells also showed a significantly increased in EGCG-treated (25 and 50 mg/kg) rats after PNCI at various time points (*p *< 0.05 vs. non-treated groups; Table 1). Quantitative analysis of labeling intensity showed stronger diaphorase reactivity in cells of non-treated rats than those in rats treated with high-dose EGCG (25 and 50 mg/kg) at 3, 7, and 14 days after PNCI (*p *< 0.05, Figure 5). However, at 28 days after PNCI, the percentage of NADPH-d(+) neurons on the crush injured side pretreated with 25 mg/kg EGCG was similar to those with 10 mg/kg EGCG or none (*p *< 0.05, Figure 4). Moreover, there was no significant decline in the total number, percentage or labeling intensity of NADPH-d/nNOS(+) neurons in rats given a low-dose (10 mg/kg) EGCG treatment (Figures 2B, E, 3B, E, 4 and 5; Table 1).

### Cell population change of in the HN and DMN after EGCG pretreatment and PNCI

In sham-operated rats irrespective of treatment, the number of motor neurons in the HN and DMN (about 1400 and 1300 cell/u, respectively) at all time points was not significantly different from that in the corresponding nuclei of the opposite side. In non-treated rats at 28 days following PNCI, about 18% and 26% neuronal loss were respectively observed in the HN and DMN (Table 2). However, in high-dose EGCG (25 and 50 mg/kg)-treated rats following PNCI, a significant attenuation of neuronal loss *(p *< 0.05, Table 2) was found when compared with those of nerve-crushed rats treated with nothing or low-dose EGCG (10 mg/kg). No contralateral (right) cell death was found in the sham-operated groups as a consequence of unilateral (left) nerve crush.

**Table 2:** Effect of EGCG treatment on the number of surviving motoneurons and percentage of cell loss in the lesioned side of DMN and HN at 3, 7, 14 and 28 days following PNCI

Days postoperative	Hypoglossal nucleus				
	
	Sham	Saline	EGCG (10 mg/kg)	EGCG (25 mg/kg)	EGCG (50 mg/kg)
3	1416 ± 11	1405 ± 8	1408 ± 9	1398 ± 8	1399 ± 9
	-	1 ± 0.6%	1 ± 0.6%	1 ± 0.8%	1 ± 0.9%
7	1408 ± 10	1294 ± 18	1304 ± 17	1360 ± 11*	1365 ± 7*
	-	9 ± 1.7%	8 ± 1.9%	4 ± 1.2%*	4 ± 1.0%*
14	1411 ± 11	1212 ± 31	1238 ± 20	1318 ± 10*	1331 ± 7*
	-	14 ± 2.0%	12 ± 2.0%	7 ± 1.0%*	6 ± 1.0%*
28	1397 ± 8	1165 ± 20	1175 ± 30	1291 ± 5*	1301 ± 5*
	-	18 ± 2.0%	17 ± 2.0%	9 ± 1.0%*	8 ± 1.0%*

**Days postoperative**	**Dorsal motor nucleus of vagus nerve**				
	
	**Sham**	**Saline**	**EGCG** **(10 mg/kg)**	**EGCG** **(25 mg/kg)**	**EGCG** **(50 mg/kg)**

3	1284 ± 10	1277 ± 13	1286 ± 8	1293 ± 8	1289 ± 7
	-	1 ± 0.5%	0 ± 0.7%	0 ± 0.6%	0 ± 0.7%
7	1292 ± 8	1182 ± 19	1185 ± 16	1227 ± 11*	1235 ± 8*
	-	8 ± 1.5%	8 ± 1.6%	4 ± 0.7%*	4 ± 0.7%*
14	1289 ± 11	1055 ± 20	1079 ± 14	1182 ± 13*	1192 ± 7*
	-	18 ± 1.3%	16 ± 1.4%	8 ± 0.7%*	7 ± 1.0%*
28	1283 ± 8	952 ± 21	983 ± 17	1021 ± 15*	1091 ± 27*
	-	26 ± 1.2%	23 ± 1.4%	20 ± 1.3%*	15 ± 2.3%*

*, *p *< 0.05 when compared with the saline-treated group

## Discussion

The present study has provided the first morphological evidence that EGCG treatment may reduce damage caused by nerve crush-induced oxidative injury in brainstem motor neurons having different functions in adult rats. The protective effect of EGCG on injured neurons was dose-dependent. Taken together, our results suggest that the protective effect of higher-dose EGCG may be responsible for suppressing PNCI-induced NADPH-d/nNOS expression in the HN and DMN by its higher antioxidant ability.

### Role of NADPH-d/nNOS expression in motor neurons after PNCI

The current study revealed induced expression of NADPH-d/nNOS in the lower brainstem motor neurons of adult rats following PNCI. At higher doses (25 and 50 mg/kg), EGCG significantly reduced the total number, percentage and enzyme activity of cells expressing NADPH-d/nNOS; this beneficial effect was more pronounced with injury progress. Ultimately, this reduction in NADPH-d/nNOS reactivity was markedly correlated with the diminution of motor neuron loss at 7, 14, and 28 days after PNCI. It is well documented that initial NO level and the time of exposure to excess NO, generated by NOS via the overstimulation of NMDA receptors, may account for neuronal damage following PNI [45,46]. NO itself or peroxynitrite (ONOO^-^), which is formed when NO couples with the superoxide anion, can trigger a series of biochemical reactions that culminate in damage to mitochondria, nucleic acids, proteins, lipids, or DNA [9,47,48]. The coincidence of overt lesion-induced NADPH-d/nNOS expression with the death of injured motor neurons has therefore been found [14,49,50]. On the other hand, inhibition of NOS production in mice following spinal root avulsion has been shown to effectively reduce neuronal death and enhance peripheral nerve regeneration [14,15,51]. Our previous and other studies on the motor neurons of adult rats have also shown that PNI along with hypoxia or PNCI significantly up-regulated NADPH-d/nNOS expression [17]. This up-regulation of enzyme expression is due to the production of excess NO that has detrimental effects on these neurons [23,27]. Interestingly, preconditioning treatment with mild hypoxic may induce small amount of NO that may alter the neuronal milieu and consequently protect against nerve crush-induced oxidative injury. Thus, whether NO is protective or destructive depends on its amount: the small amount would protect against neuronal damage and the large one cause cell death. In the present study, moderate and transient NADPH-d/nNOS expression was observed in the majority of HN neurons with PNCI progression during which mild neuronal death occurred. By contrast, NADPH-d/nNOS expression in DMN motor neurons after PNCI was prolonged and cell loss was pronounced with injury development (Figures 2, 3, and 7; Table 1 and 2). These results implied a close association of NADPH-d/nNOS expression with progressive neuronal death following PNCI. However, in lack of experimental evidence, the assumption that NADPH-d/nNOS positive neurons were the cells that died after the nerve injury may not necessarily be the case. The changes of NADPH-d or NOS activity may be part of a dynamic response to injury rather than simply pathologically related.

### Effects of EGCG on NADPH-d/nNOS expression in motor neurons following PNCI

Our previous and other studies have shown that green tea, melatonin, and hypoxic preconditioning strategies may enhance endogenous antioxidative defense systems against oxidative injury [20,22,23,26-28,52]. Green tea, rich in polyphenols, is one of the most widely consumed beverages in the world. EGCG is the most abundant catechin in tea and the major source of polyphenol bioactivity. Several studies have indicated that the antioxidant activity of EGCG may be neuroprotective and prevent NO-mediated neurological deficits. In *in vitro *studies, EGCG can scavenge cytotoxic NO or its downstream products [35,53]. The ability of EGCG to protect against neuronal damage and brain edema after unilateral cerebral ischemia and to reduce hippocampal neuronal damage after transient global ischemia in gerbils has been evidenced [54,55]. Our previous report also showed that EGCG may impart the suppressive effect on susceptibility of nodose neurons to nNOS-mediated neuropathy subsequent to severe hypoxic exposure [26]. It has been suggested that NO produced by nNOS and iNOS plays an important role in pathophysiologic processes including neurotoxicity and delayed neuronal death [47,56]. Consequently, the amount of NOS reactivity may be used as an index of neuronal damage severity. The present results clearly indicated that high-doses EGCG (25 and 50 mg/kg) reduced NADPH-d/nNOS expression and neuronal loss following PNCI that caused excessive and prolonged expression of NADPH-d/nNOS leading to neurodestruction in the region studied. Reducing the activity of this enzyme (e.g., by treatment with EGCG) could decrease neurological signs and neuronal damage as reflected by evidences of our previous and other studies. All these results showed that nNOS inhibitors, melatonin, and high-doses EGCG (25 and 50 mg/kg) reduced nNOS activity and neuronal damage after brain ischemia, hypobaric hypoxia, and PNI [20,26,54,57]. Similarly, the present study found that high-doses EGCG (25 and 50 mg/kg) may be neuroprotective and thereby attenuate neuronal damage after peripheral nerve crush injury.

### Possible mechanisms regarding the differential responses of different motor neurons to EGCG treatment after PNCI

Our previous and other studies found differences in PNI-induced NADPH-d/nNOS expression between motor neurons having different functions, namely, somatic efferents of the HN and visceral efferents of the DMN [23,27]. In agreement with these findings, the present results demonstrated a peak of PNCI-induced NADPH-d/nNOS expression in motor neurons of the HN at 7 days, whereas those of the DMN persisted up to 28 days (Figures 2, 3 and 4). This difference between the motor neurons of the HN and DMN remains to be resolved but may be due to differences in their regenerative capacity. Previous tracing study with horseradish peroxidase demonstrated that the majority of axotomized hypoglossal motor neurons re-innervated tongue muscles at 14 days post-PNI [16]. During re-innervation, neurotrophic factors may stimulate motor neuron regeneration and thereby ensure the decrease in NADPH-d/nNOS expression in successful regenerating motor neurons [17,58,59]. Thus, to the extent that NADPH-d/nNOS expression is correlated with the degree of functional recovery by injured motor neurons, down-regulation of NADPH-d/nNOS expression indicates a predictable functional recovery of motor neurons in the crushed HN. Furthermore, the suppressive NADPH-d/nNOS expression seen in rats treated with high-doses EGCG (25 and 50 mg/kg) at all time points post-PNI implied that high-dose EGCG facilitated functional recovery of the damaged HN. In contrast, the current investigation also found that the sustained expression of NADPH-d/nNOS in damaged motor neurons of the DMN adversely affected their functional recovery [17]. Our present evidences may echo the finding that axonal regeneration appeared to be absent in visceral motor neurons of the DMN [17]. However, in view of the fact that the viability of surviving motor neurons following PNI depended on their proximity to injured axons [51,60], the injury sites of the hypoglossal and vagus nerves should be seriously considered. In our present study, the nerve crush at the cervical level of the vagus nerve relative to that of the hypoglossal nerve may have been proximal enough to cause severe damage to DMN motor neurons.

## Conclusion

The current study demonstrated morphologically and quantitatively that high-doses (25 and 50 mg/kg) EGCG can reduce PNCI-induced NADPH-d/nNOS expression in the HN and DMN motor neurons. This novel finding not only improved our understanding of the functional role of NO in neuronal damage, but also provided an EGCG neuroprotective strategy to suppress nNOS-mediated toxicity in the HN and DMN motor neurons damaged by PNCI.

## Authors' contributions

IHW, CCH, MHT and JYS conceived this experiment. Animal studies were performed by IHW, HCT and CYT. Immunohistochemistry was performed by IHW, HCT, MHT and CYT. Data acquisition, analysis and manuscript preparation were performed by IHW, HCT, CCH, MHT and JYS. The manuscript was finally edited by IHW, CCH, MHT and JYS. All authors had read and approved the final submitted and published versions.
